# Coverage and Timeliness of Birth Dose Vaccination in Sub-Saharan Africa: A Systematic Review and Meta-Analysis

**DOI:** 10.3390/vaccines8020301

**Published:** 2020-06-11

**Authors:** Oumar Bassoum, Moe Kimura, Anta Tal Dia, Maud Lemoine, Yusuke Shimakawa

**Affiliations:** 1 Faculté de Médecine, de Pharmacie et d’Odontologie, Université Cheikh Anta Diop de Dakar, Dakar-Fann 5005, Senegal; bassoum.oumar@gmail.com (O.B.); anta.dia@ucad.edu.sn (A.T.D.); 2 Institut de Santé et Développement, Université Cheikh Anta Diop de Dakar, Dakar-Fann 16390, Senegal; 3 Department of Metabolism, Digestion and Reproduction, Section of Hepatology and Gastroenterology, Imperial College, London W2 1NY, UK; moekimura123@gmail.com (M.K.); m.lemoine@imperial.ac.uk (M.L.); 4 Faculty of Medicine, Tokyo Medical and Dental University, Tokyo 113-8510, Japan; 5 Unité d’Épidémiologie des Maladies Émergentes, Institut Pasteur, 25-28 rue du Dr Roux, 75015 Paris, France

**Keywords:** birth dose vaccination, BCG, polio vaccine, hepatitis B vaccine, sub-Saharan Africa, systematic review

## Abstract

*Background*: Depending on the epidemiological context of each country, three vaccines are recommended by the World Health Organization (WHO) to be administered as soon as possible after birth (birth vaccines); namely, BCG, zero dose of oral polio vaccine (OPV0), and birth dose of hepatitis B vaccine (HepB-BD). The timely administration of these vaccines immediately after birth might pose significant challenges in sub-Saharan Africa, where about half of childbirths occur outside health facilities. We therefore conducted a systematic review and meta-analysis to estimate the coverage rate of these vaccines at a specific timing in neonates in sub-Saharan Africa. *Methods*: We searched PubMed, Embase, CINAHL, and Web of Science for studies conducted in sub-Saharan Africa and published up to March 31, 2017, which provided a coverage rate of the birth vaccines at any specific time points within 28 days after birth. Two investigators independently screened the titles and abstracts and extracted data from the eligible full-text articles. This study was registered in PROSPERO (CRD42017071269). *Results*: Of 7283 articles identified, we finally included 31 studies with 204,111 infants in the meta-analysis. The pooled coverage rates at day 0–1 after birth were 14.2% (95% CI: 10.1–18.9) for BCG and 1.3% (0.0–4.5) for HepB-BD. No data were available for OPV0 at day 0–1. The coverage at day 28 was 71.7% (63.7–79.2) for BCG, 60.8% (45.8–74.7) for HepB-BD, and 76.1% (67.1–84.0) for OPV0. No significant difference in the vaccine coverage was observed between infants born in healthcare facilities and those born outside facilities. *Conclusions*: The rates of vaccine coverage immediately after birth were very low for BCG and HepB-BD, and no data for OPV0. We need additional data to better define barriers and facilitators for the timely administration of the birth vaccines in sub-Saharan Africa, since the delay in its provision may increase the burden of these vaccine-preventable diseases.

## 1. Introduction

Vaccination is a highly cost-effective intervention to reduce morbidity and mortality on a global scale [1]. It is estimated that childhood vaccination prevents more than two million deaths annually [2]. Depending on the epidemiological context of each country, there are three vaccines that are recommended by the World Health Organization (WHO) to be administered as soon as after birth (birth dose vaccines), namely, Bacillus Calmette-Guérin (BCG), zero dose of oral polio vaccine (OPV0), and birth dose of hepatitis B vaccine (HepB-BD). Of these, HepB-BD is explicitly indicated to be given within 24 h after birth, while the other two vaccines do not have such a precision [3,4,5]. Administration of these vaccines immediately after birth aims at reducing pediatric tuberculosis mortality for BCG [3,6], increasing the levels of neutralizing antibodies against poliovirus and sero-conversion rates with completion of subsequent doses for OPV [4], and preventing both perinatal mother-to-child and early horizontal transmission of hepatitis B virus (HBV) for HepB-BD [5]. In addition, accumulating evidence supports the nonspecific effects of BCG or OPV0 administered at birth on overall childhood mortality that is not explained by preventing the diseases targeted by these vaccines [7,8].

Sub-Saharan Africa is a region with the highest risk of infant mortality; 53 out of 1000 births die within the first month of life in 2018 [9]. Among the 48 countries in sub-Saharan Africa, BCG and OPV0 are scheduled at birth in 45 and 39 countries, respectively, whilst only 12 countries have introduced HepB-BD in their national immunization programs [10]. According to the WHO/UNICEF estimates in 2018, the coverage of BCG, third dose of OPV, and HepB-BD in infants in the WHO African region was 80%, 74% and 4%, respectively [11]. However, these proportions do not consider whether the vaccination was given at birth, particularly for BCG and OPV. The BCG coverage is based on the number of vaccinations in a specific year divided by the number of live births [12]. The coverage of OPV is based on the number of children who have completed its 3rd dose; no specific estimate is made for the coverage of OPV0. For HepB-BD, the coverage data have only recently been improved by distinguishing whether the vaccine was given within or after 24 h of birth [13]. We, therefore, conducted a systematic review and meta-analysis to estimate the coverage and determinants of the timely administration of birth-dose vaccination in sub-Saharan Africa.

## 2. Materials and Methods

This systematic review followed a protocol registered at PROSPERO (CRD42017071269) and was reported according to the Preferred Reporting Items for a Systematic Review and Meta-Analysis (PRISMA) guidelines [14].

### 2.1. Data Sources and Searches

The following four databases were searched for articles published up to 31st March 2017: PubMed, EMBASE, CINAHL, and Web of Science. The search strategy was structured around three concepts: (i) BCG, OPV, or hepatitis B vaccine, AND (ii) vaccination coverage, AND (iii) sub-Saharan Africa. The detailed search strategy is presented in the Supplementary Document 1. There was no restriction on any language.

### 2.2. Eligibility Criteria

We included both observational and intervention studies which provided an estimate of the coverage of the birth vaccines at specific time points in sub-Saharan Africa. We defined BCG, OPV0, and HepB-BD as the birth vaccines. The vaccination status of each individual needed to be ascertained through written documents including a vaccination card or registry. We did not consider studies that ascertained vaccination status solely through caregiver’s recalls. We excluded studies that only reported the coverage beyond 28 days.

### 2.3. Data Extraction

Two authors (O.B. and K.M.) independently screened the titles and abstracts of articles identified from the database search. Disagreements were resolved by the third reviewer (Y.S.). For potentially eligible articles, full-text papers were obtained and independently processed for data extraction by two authors (O.B. and K.M.). For each selected study, a pre-piloted standardized form was used to extract the following data: author names, year, country, study area (urban or rural), study design, study settings (hospital-based or population-based), source of vaccination information (vaccination cards, registries at health facilities, others), percentage of study participants having a valid source of vaccination information (percentage ascertained), children’s age at the coverage assessment, maternal characteristics (age, HIV infection status, education level), place of birth, birth weight, type of vaccine, timing assessed for vaccination coverage, and percentage of neonates receiving each vaccine at each time point. Following the WHO’s instruction, we considered the timely administration within 24 h after birth if the vaccine was given within the first day of life (i.e., vaccine given on day 0 or day 1) [13]. Corresponding authors of the articles were contacted whenever essential information was missing. The risk of bias was independently examined by two authors using a standardized checklist which was adapted from the framework presented by Altman (Supplementary Document 2) [15].

### 2.4. Statistical Analysis

Our primary outcome of interest was the proportion of infants who have received each type of the birth vaccines by certain time points within 28 days after birth. We obtained this by dividing the number of infants vaccinated by the specific time point by the total number of infants assessed. We excluded infants without a valid source of vaccination information from the denominator of these analyses. To pool the proportions, we carried out a meta-analysis using a command ‘metaprop’ with STATA 16.0 (STATA Corporation, College Station, TX, USA) [16]. We stabilized the variance of the proportions using Freeman–Tukey double arcsine transformation [17], then, pooled these estimates using the DerSimonian–Laird random-effects model [18]. We computed the confidence intervals of the individual studies using the score test, and those of the pooled estimates using the Wald test [16]. We examined the percentage of total variation between the studies due to heterogeneity using the I^2^ statistic [7]. To assess determinants associated with coverage and timeliness of vaccines, we conducted a subgroup analysis for the following variables that were determined *a priori*: study location (rural or urban); place of birth (at health facilities or outside); birth weight; maternal education level; and maternal age. We reported these subgroup analyses irrespective of the type of the birth vaccines.

## 3. Results

### 3.1. Study Selection

**Figure 1:**
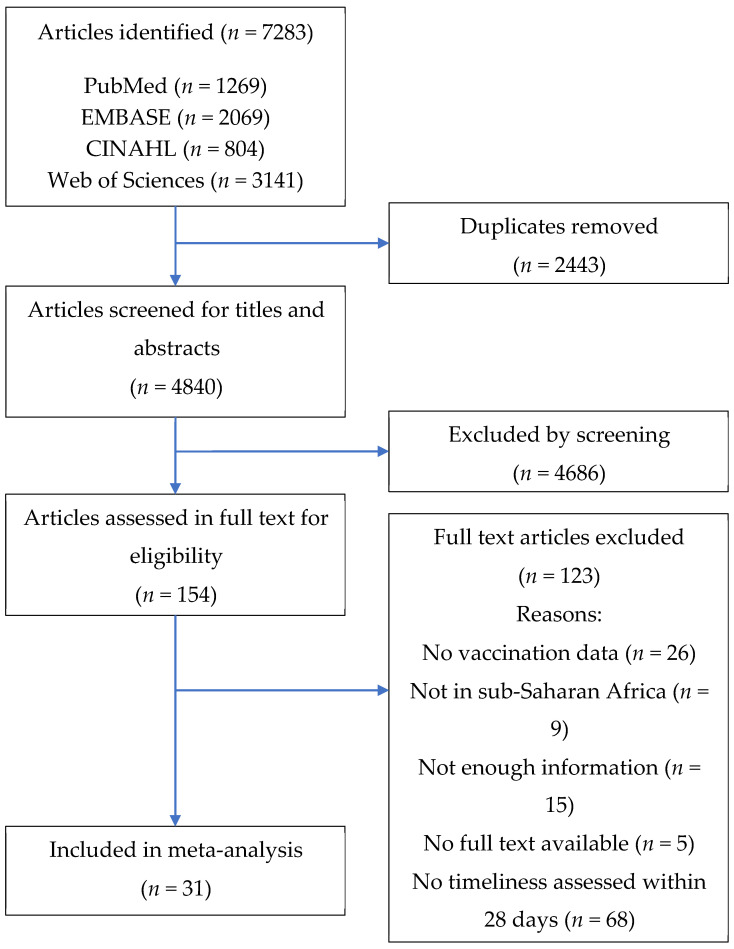
Flow diagram of study selection.

A total of 7283 articles were identified from the search of four databases. After duplicates have been removed, 4840 articles were screened, and 154 articles were selected for full-text readings. A total of 123 articles were removed after full-text readings by the following reasons: not assessing BCG, OPV0 or HepB-BD (*n* = 26); not in sub-Saharan Africa (*n* = 9); not enough information (*n* = 15); no full text available (*n* = 5); or not assessing timeliness within 28 days of birth (*n* = 68). Finally, 31 articles were included in the meta-analysis (Figure 1).

### 3.2. Study Characteristics

**Figure 2:**
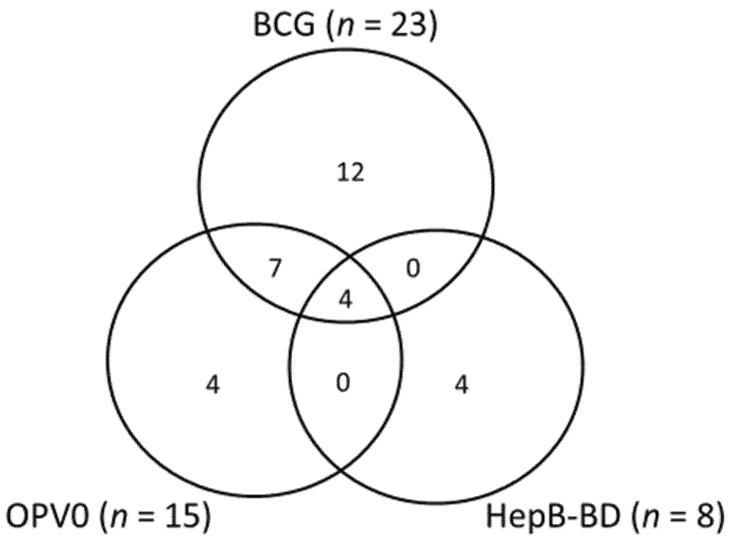
Venn diagram showing the number of studies evaluated HepB-BD, BCG, and OPV0.

The characteristics of the included studies are summarized in Table 1. Among the 31 studies, 20 were cross-sectional design, 7 were prospective cohort studies, and 4 were retrospective cohort studies. There were 15 studies from West Africa (Burkina Faso (*n* = 1), The Gambia (*n* = 2), Ghana (*n* = 3), Guinea Bissau (*n* = 2), and Nigeria (*n* = 7)), one study from Central Africa (Democratic Republic of Congo (*n* = 1)), 12 studies from East Africa (Ethiopia (*n* = 1), Kenya (*n* = 5), Malawi (*n* = 1), Tanzania (*n* = 2) and Uganda (*n* = 3)), and 3 studies from Southern Africa (South Africa (*n* = 3)). There was one study that integrated results of Demographic Health Surveys from 29 countries across sub-Saharan Africa [19]. Out of the 31 studies, 23 used vaccination cards as the primary source of vaccination status, five were based on clinical records, and three used both of these as the information sources. The largest study using the data from Demographic Health Surveys ascertained BCG vaccination status through verification of infants’ vaccination cards [19]. The percentage ascertained, defined by the proportion of the study participants who had a valid source of vaccination information, ranged from 27.3% to 99.8% with a median of 85.0% (Table 1). The number of studies assessing BCG, OPV0, and HepB-BD was 23, 15, and 8, respectively (Figure 2). There was a wide variation in the time points assessed in the included studies: at day 0–1 (*n* = 5), day 7 (*n* = 13), day 14 (*n* = 13), and day 28 (*n* = 17).

### 3.3. Risk of Bias

A detailed description of the risk of bias of each of the included studies was presented in the Supplementary Document 3. A great majority of the studies provided sufficient information for eligibility criteria, patient sampling, and methods to collect outcomes. The completeness of the study, defined by the proportion of the eligible persons who participated in the study, was reported in 13 studies and ranged between 63.9% and 100%.

### 3.4. Coverage and Timeliness of Birth Dose Vaccines

**Figure 3:**
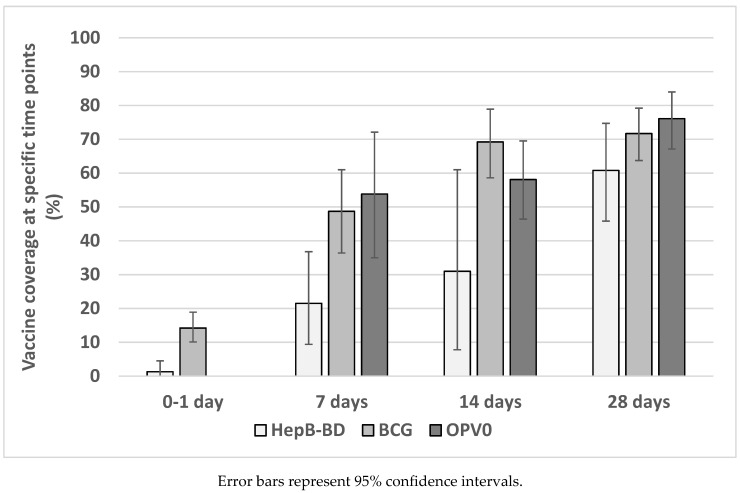
Pooled estimates of birth vaccine coverage at specific age.

**Figure 4:**
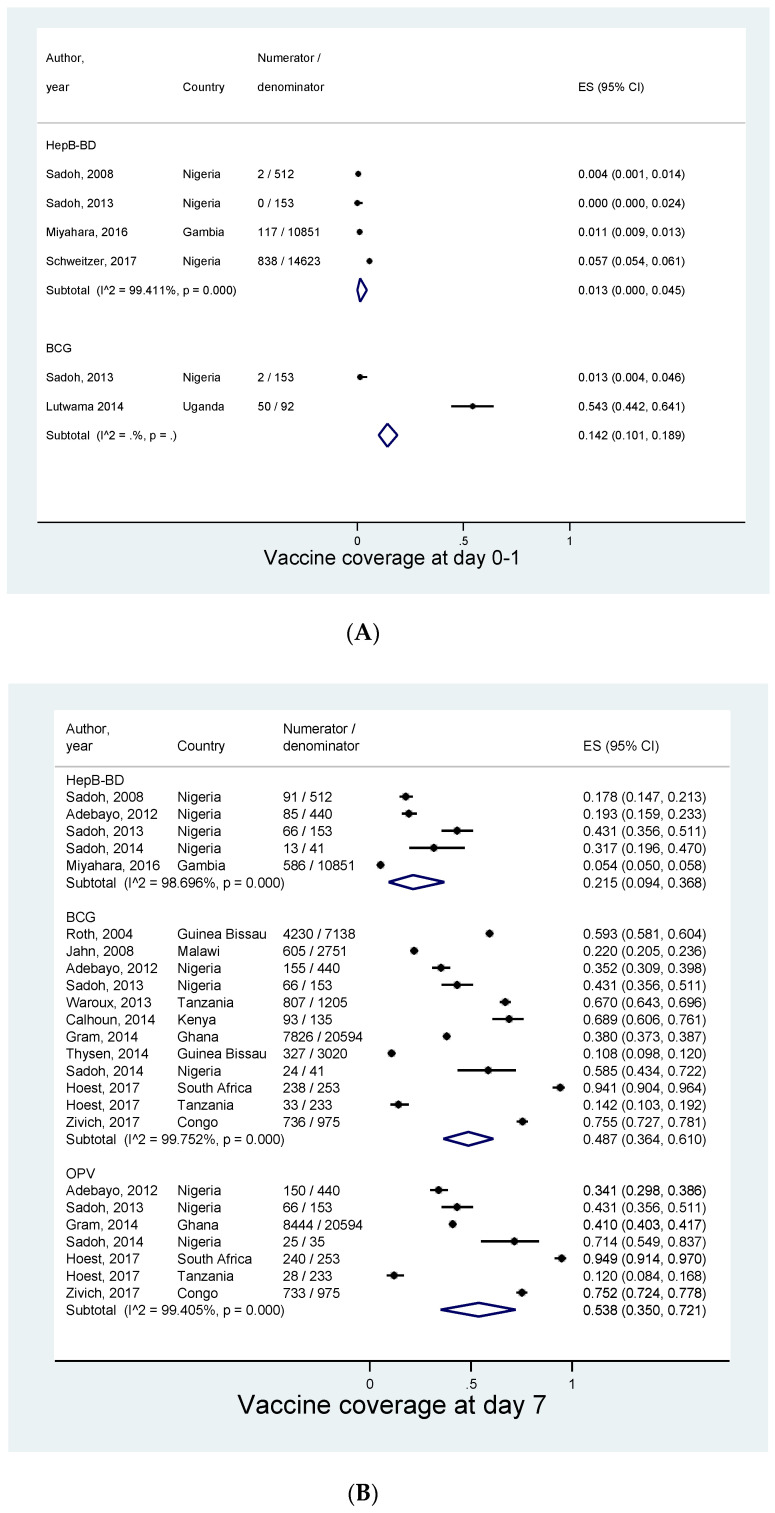

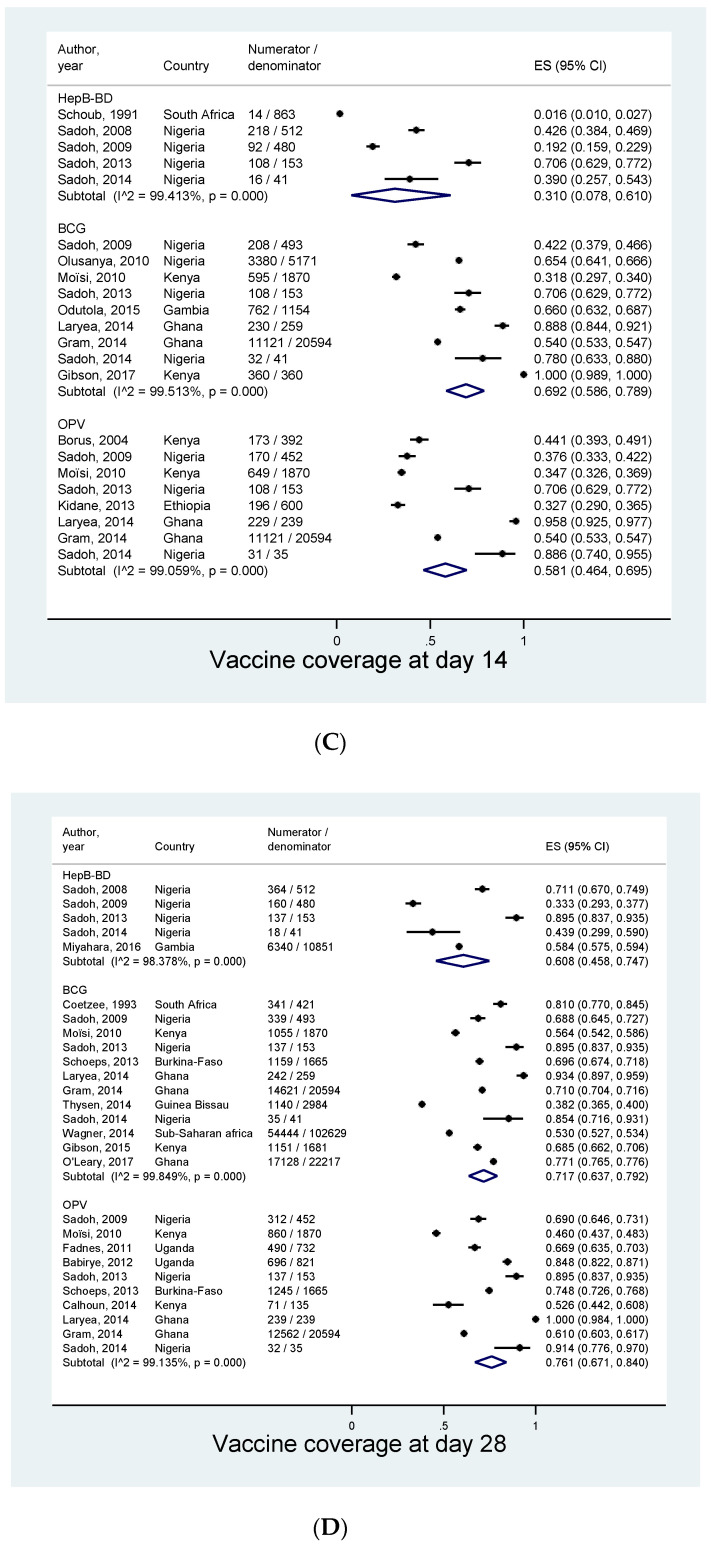
Coverage of birth vaccines at different time points. (**A**) At birth (0–1 day). (**B**) At day 7. (**C**) At day 14. (**D**) At day 28.

A total of 204,111 children from 31 studies were included in the meta-analysis. Meta-analysis could be performed at four different time points: day 0–1, day 7, day 14, and day 28. The pooled coverages increased with an increase in infants’ age (Figure 3). Five studies provided data for a coverage at day 0–1 after birth. The pooled coverage of BCG and HepB-BD was 14.2% (95% Cl: 10.1–18.9%) and 1.3% (0.0–4.5%), respectively (Figure 4A). None of the included studies provided an estimate for the OPV0 coverage at day 0–1. The pooled coverage at day 7 was 48.7% (95% CI: 36.4–61.0%) for BCG, 53.8% (35.0–72.1%) for OPV0, and 21.5% (9.4–36.8%) for HepB-BD (Figure 4B). The pooled coverage at day 14 was 69.2% (95% CI: 58.6–78.9%) for BCG, 58.1% (46.4–69.5%) for OPV0, and 31.0% (7.8–61.0%) for HepB-BD (Figure 4C). Finally, the coverage at day 28 was 71.7% (95% CI: 63.7–79.2%) for BCG, 76.1% (67.1–84.0%) for OPV0, and 60.8% (95% CI: 45.8–74.7%) for HepB-BD (Figure 4D). In most of the meta-analyses there was very strong evidence for heterogeneity across the studies (I^2^ >98%, *p* < 0.001).

### 3.5. Subgroup Analysis

**Figure 5:**
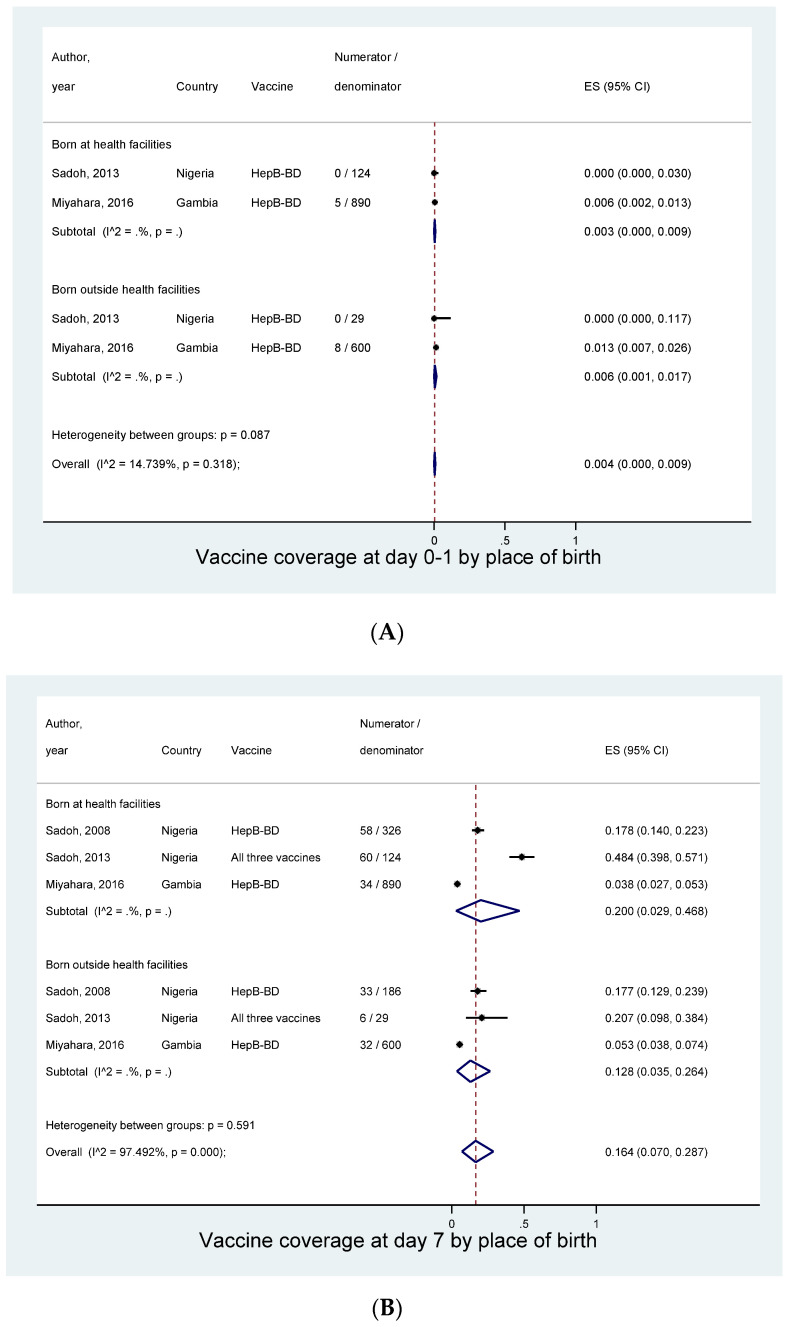

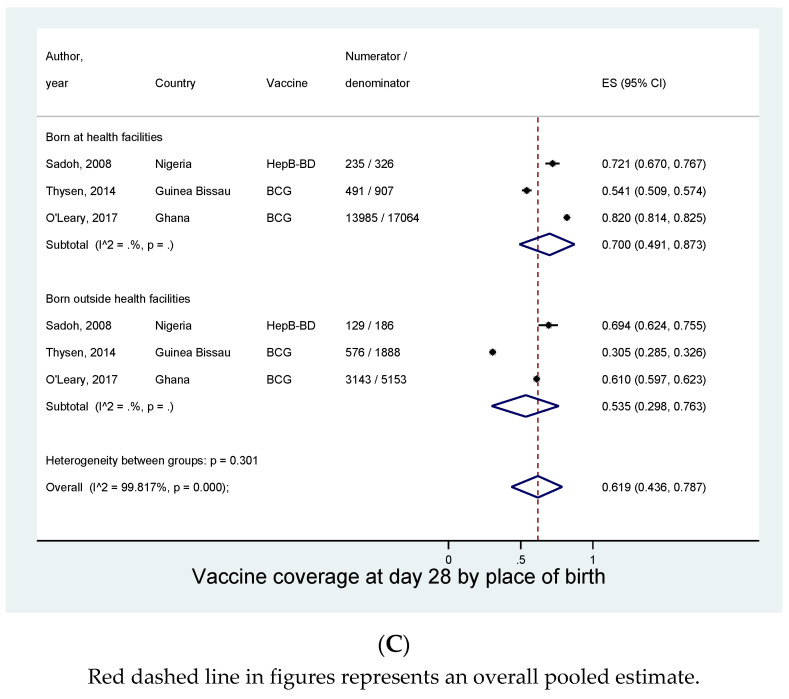
Coverage of birth vaccines by place of birth. (**A**) At birth (0–1 day). (**B**) At day 7. (**C**) At day 28.

Given the limited number of the studies at each specific time points, we could conduct a subgroup analysis only for the “place of birth”. Irrespective of the type of vaccines we assessed whether vaccine coverage differed by where the infants were born. At day 0–1, the coverage was 0.3% (95% CI: 0.0–0.9%) in those born at health facilities and 0.6% (0.1–1.7%) in those born outside health facilities (Figure 5A). At day 7, infants born in health facilities were more likely to receive birth vaccines (20.0%, 95% CI: 2.9–46.8%) when compared to infants born outside health facilities (12.8%, 3.5–26.4%), although the difference was not significant (*p* = 0.5) (Figure 5B). Such a non-significant trend was also observed at day 28: 70.0% (95% Cl: 49.1–87.3%) in those born in facilities and 53.5% (29.8–76.3%) in those born outside facilities (*p* = 0.3) (Figure 5C).

## 4. Discussion

In this systematic review and meta-analysis evaluating the timeliness of the birth vaccines in sub-Saharan Africa, the pooled rates of vaccine coverage at the age of four weeks were modest: BCG (71.7%), OPV0 (76.1%), and HepB-BD (60.8%). However, the pooled rates at birth within the first date of life were far from satisfaction: BCG (14.2%) and HepB-BD (1.3%). The coverage on day 7 was still low, with more than half of the infants remaining unvaccinated. In addition, in most of these meta-analyses, we identified strong evidence for heterogeneity across the studies. We expected such a wide geographical and temporal variation in the vaccine coverages because of the social, cultural, economic, and political differences between the contexts of the included studies in terms of vaccination practices.

The timely administration is critical in conferring the expected effects of the birth vaccines to the children. BCG given shortly after birth is especially important in areas with a high incidence of tuberculosis where there is an elevated risk of early exposure to mycobacterium tuberculosis [3]. It is known that the zero dose of OPV significantly improves the seroconversion rate to poliovirus following the completion of the subsequent doses [4]. Mother-to-child transmission of HBV is known to be associated with a high risk of developing chronic HBV infection, and also progressing towards chronic liver diseases including cirrhosis and liver cancer [50,51]. Since the majority of mother-to-child transmission occurs perinatally through contact with maternal body fluid, HepB-BD needs to be given as soon as possible after birth [52]. In addition to failing to receive timely protection from these preventable diseases, infants not vaccinated according to the recommended schedule may have an increased risk of never fully completing a whole series of the subsequent vaccines [53].

Our study highlighted the limited number of studies in the region assessing the coverage of birth vaccines, suggesting a potentially low interest and commitment to the timely birth dose vaccination in sub-Saharan Africa. Although administration as soon as after birth is recommended by the WHO, only five included studies focused on this time point. Moreover, the number of studies assessing HepB-BD was far more limited compared to the other two vaccines. In Africa, only 12 out of 48 countries integrated HepB-BD in the routine infant immunization schedule [54]. Therefore, the low coverage observed in this meta-analysis, which was only derived from the data from the countries that had already integrated HepB-BD in the national program, should be an overestimate of the actual situation in sub-Saharan Africa as a whole region, since the majority of infants are born in countries without any access to HepB-BD.

Timely administration of birth vaccines is especially challenging in sub-Saharan Africa where about half of women give birth at home [55,56]. Indeed, in our subgroup analysis on day 7 and day 28, there was a non-significant trend that children born outside health facilities were less likely to receive birth vaccines than those born at facilities, which was consistent with previous reports [38,41,45,46]. Without providing a special mechanism to deliver vaccines at home, such as outreach immunization strategy, it is difficult to vaccinate babies born at home immediately after their birth [57]. In contrast, high coverage at birth should be expected for those born at healthcare facilities if the birth vaccines are well integrated into maternity and postnatal care. Nevertheless, we found the very low vaccine coverage at birth (day 0–1) even in those born at health facilities: 0% (0/124) for HepB-BD and 1.6% (2/124) for BCG in Sadoh’s study in Nigeria; and 0.6% (5/890) for HepB-BD in Miyahara’s study in The Gambia [28,32]. Sadoh et al., explained that in Nigeria this low coverage was due to limited awareness of the birth vaccines among the healthcare workers, and poor communication with mothers. Moreover, BCG is often scheduled at a fixed date to avoid any wastage of unused vaccine in multi-dose vials [46]. These underline the importance of elucidating barriers to timely birth dose vaccination within health facilities in sub-Saharan Africa.

Our study has several limitations. Since there was a wide variation in time points to assess vaccine coverage according to the different studies, the number of studies at each time point was limited. Of six *a priori* sources of heterogeneity, we could only assess “place of birth” in the subgroup analyses. A great majority of the included studies did not report the completeness of the study population; care must be therefore taken to interpret the results in terms of representativeness of the study population. Moreover, we excluded study participants without a valid source of vaccination information from our meta-analyses. Since these infants who do not possess the vaccination card at the time of a survey are less likely to have received the vaccine in a timely manner, we might have over-estimated the coverage at each specific time point. Finally, this was a meta-analysis of aggregate data, pooling cumulative incidences of being vaccinated by the certain time points. We therefore could not assess the exact timing of vaccine administration in each individual.

## 5. Conclusions

Although the majority of neonates received the birth vaccines by the first month of life, only a few received them at birth. We need additional data to better define barriers and facilitators for the administration of these vaccines at birth in sub-Saharan Africa, and this may allow us to develop evidence-based interventions to improve the coverage and timeliness of these life-saving vaccines.

## Figures and Tables

**Table 1:** Characteristics of the included studies.

Author, Year, Reference	Country	Study Design	Study Setting	% Ascertained	Vaccine Assessed	Time Points Assessed (days)	Total No. of Infants Assessed
**Studies using vaccination cards only**							
Coetzee, 1993 [20]	South Africa	Cross-sectional	Population	88.0%	BCG	28	421
Roth, 2004 [21]	Guinea Bissau	Retrospective cohort	Hospital	84.2%	BCG	7	7138
Jahn, 2008 [22]	Malawi	Cross-sectional	Population	78.0%	BCG	7	2751
Moisi, 2010 [23]	Kenya	Cross-sectional	Population	86.2%	BCG, OPV0	14/28	1870
Fadnes, 2011 [24]	Uganda	Prospective cohort	Hospital	98.0%	OPV0	28	732
Adebayo, 2012 [25]	Nigeria	Cross-sectional	Population	N/R	BCG, OPV0, HepB-BD	7	440
Babirye, 2012 [26]	Uganda	Cross-sectional	Population	91.2%	OPV0	28	821
Schoeps, 2013 [27]	Burkina Faso	Cross-sectional	Population	N/R	BCG, OPV0	28	1665
Waroux, 2013 [28]	Tanzania	Cross-sectional	Population	59.0%	BCG	7	1205
Calhoun, 2014 [29]	Kenya	Retrospective cohort	Population	55.3%	BCG, OPV0	7/28	135
Gram, 2014 [30]	Ghana	Prospective cohort	Population	N/R	BCG, OPV0	7/14/28	20,594
Laryea, 2014 [31]	Ghana	Cross-sectional	Hospital	N/R	BCG, OPV0	14/28	259
Sadoh, 2014 [32]	Nigeria	Cross-sectional	Hospital	27.3%	BCG, OPV0, HepB-BD	7/14/28	41
Thysen, 2014 [33]	Guinea Bissau	Cross-sectional	Population	85.0%	BCG	7/28	3020
Wagner, 2014 [19]	Sub-Saharan Africa *	Cross-sectional	Population	N/R	BCG	28	102,629
Odutola, 2015 [34]	The Gambia	Cross-sectional	Hospital	N/R	BCG	14	1154
Lutwama 2014 [35]	Uganda	Cross-sectional	Hospital	N/R	BCG	0–1	92
Gibson, 2015 [36]	Kenya	Cross-sectional	Population	63.9%	BCG	28	1681
Miyahara, 2016 [37]	The Gambia	Cross-sectional	Population	99.8%	HepB-BD	0–1/7/28	10,851
O’Leary, 2017 [38]	Ghana	Prospective cohort	Population	96.5%	BCG	28	22,217
Gibson, 2017 [39]	Kenya	Prospective cohort	Population	N/R	BCG	14	360
Schweitzer, 2017 [40]	Nigeria	Cross-sectional	Population	N/R	HepB-BD	0–1	14,623
Zivich, 2017 [41]	Congo	Prospective cohort	Hospital	93.5%	BCG, OPV0	7	975
**Studies using clinical records only**							
Schoub, 1991 [42]	South Africa	Prospective cohort	Hospital	N/R	HepB-BD	14	863
Sadoh, 2008 [43]	Nigeria	Retrospective cohort	Hospital	N/R	HepB-BD	0–1/7/14/ 28	512
Sadoh, 2009 [44]	Nigeria	Retrospective cohort	Population	N/R	BCG, OPV0, HepB-BD	14/28	493
Olusanya, 2010 [45]	Nigeria	Cross-sectional	Hospital	68.9%	BCG	14	5171
Sadoh, 2013 [46]	Nigeria	Cross-sectional	Hospital	N/R	BCG, OPV0, HepB-BD	0–1/7/14/ 28	153
**Studies using both vaccination cards and clinical records**							
Borus, 2004 [47]	Kenya	Cross-sectional	Hospital	N/R	OPV0	14	392
Kidane, 2013 [48]	Ethiopia	Cross-sectional	Population	N/R	OPV0	14	600
Hoest, 2017 [49]	South Africa and Tanzania	Prospective cohort	Population	N/R	BCG, OPV0	7	253

* This study combined data from the Demographic Health Surveys (DHS) conducted in 29 countries in sub-Saharan Africa.

## Supplementary Materials

The following are available online at https://www.mdpi.com/2076-393X/8/2/301/s1; Supplementary Document 1: Search strategy for PubMed; Supplementary Document 2: Risk of bias examined; Supplementary Document 3: Risk of bias for included studies.
