# Supplementary material for: Coverage and Timeliness of Birth Dose Vaccination in Sub-Saharan Africa: A Systematic Review and Meta-Analysis
Source: Vaccines (Basel). 2020 Jun 11;8(2):301. doi: 10.3390/vaccines8020301 (PMC7350240; doi:10.3390/vaccines8020301)
Supplement: Supplementary file 1 [file vaccines-08-00301-s001.pdf]

Coverage and timeliness of birth dose vaccination in sub-Saharan Africa: a systematic review and meta-analysis

Supplementary Material

Contents

Supplementary Document 1. Search strategy for Pubmed 2

Supplementary Document 2. Risk of bias examined 4

Supplementary Document 3. Risk of bias for included studies 5

Supplementary Document 1. Search strategy for Pubmed

31 March 2017

Search 1
“hepatitis B vaccines”[MeSH] OR hb vaccin*[Text] OR “poliovirus vaccines”[MeSH] OR opv[Text] OR “tuberculosis vaccines”[MeSH] OR bcg[Text] OR Bacillus Calmette Guerin[Text]
N=44100

Search 2
“vaccines”[MeSH] OR “immunization”[MeSH] OR “immunization programs”[MeSH] OR vaccine*[Text] OR vaccinat*[Text] OR immuniz*[Text] OR immunis*[Text]
N=414202

Search 3
“hepatitis b”[MeSH] OR “hepatitis b virus”[MeSH] OR hepatitis b[Text] OR type b hepatitis[Text] OR hepatitis type b[Text] OR hbv[Text] OR hep b[Text] OR “poliomyelitis”[MeSH] OR “poliovirus”[MeSH] OR polio*[Text] OR “tuberculosis”[MeSH] OR “mycobacterium tuberculosis”[MeSH] OR tuberculosis[Text] OR tb[Text]
N=363020

Search 4
coverage[Text] OR uptake[Text] OR rate[Text] OR dropout[Text] OR compliance[Text] OR adherence[Text] OR completeness[Text] OR acceptance[Text] OR acceptability[Text] OR hesitancy[Text] OR timely[Text] OR timeliness[Text] OR delay[Text]
N=2729448

Search 5
“Africa South of the Sahara”[MeSH] OR Africa*[Text] OR SSA[Text] OR Angola*[Text] OR Benin*[Text] OR Botswana*[Text] OR Burkina Faso*[Text] OR Burkinabe*[Text] OR Burundi*[Text] OR Cameroon*[Text] OR Cabo Verde*[Text] OR Cape Verde*[Text] OR Central African*[Text] OR Chad*[Text] OR Comoros*[Text] OR Comoran*[Text] OR Comorian*[Text] OR Congo*[Text] OR Djibouti*[Text] OR Equatorial Guinea*[Text] OR Eritrea*[Text] OR Ethiopia*[Text] OR Gabon*[Text] OR Gambia*[Text] OR Ghan*[Text] OR Guinea*[Text] OR Guinea Bissau*[Text] OR Ivory Coast*[Text] OR Cote

d'Ivoire*[Text] OR Ivorian*[Text] OR Kenya*[Text] OR Lesotho*[Text] OR Basotho*[Text] OR Liberia*[Text] OR Madagascar*[Text] OR Malagasy*[Text] OR Malawi*[Text] OR Mali*[Text] OR Mauritania*[Text] OR Mauritius*[Text] OR Mauritian*[Text] OR Mozambi*[Text] OR Namibia*[Text] OR Niger*[Text] OR Rwanda*[Text] OR Sao Tome and Principe*[Text] OR Senegal*[Text] OR Seychell*[Text] OR Sierra Leone*[Text] OR Somali*[Text] OR South Africa*[Text] OR Sudan*[Text] OR Swazi*[Text] OR Tanzania*[Text] OR Togo*[Text] OR Uganda*[Text] OR Zambia*[Text] OR Zimbabwe*[Text]
N=1055811

Search 6
2 AND 3
N=45404

Search 7
1 OR 6
N=62924

Search 8
7 AND 4 AND 5
N=1269

Supplementary Document 2. Risk of bias examined

Adapted from the framework presented by Altman (Egger M, Smith GD, Altman D, eds. Systematic Reviews in Health Care: Meta-analysis in Context. Wiley-Blackwell; 2001).

Study feature	Qualities sought	Assessment			
1. Sample of patients	Eligibility criteria defined	Good	Poor	N/R	N/A
	Sample selection explained (setting, locations and periods of recruitment)	Good	Poor	N/R	N/A
	Demographic socio-economic characteristics fully described (maternal age, maternal education, family's SES, rural/urban residence, etc)	Good	Poor	N/R	N/A
	Representative of study population	Good	Poor	N/R	N/A
	Completeness (of the infants eligible for the study, how much proportion (%) were included)	>80%	60-79%	<60%	N/R
2. Exposure	Methods to collect exposure variables well defined	Good	Poor	N/R	N/A
	Exposure assessor blinded to outcome status	Good	Poor	N/R	N/A
3. Outcome	Methods to collect outcome variables well defined	Good	Poor	N/R	N/A
	Outcome assessor blinded to exposure status	Good	Poor	N/R	N/A

Supplementary Document 3. Risk of bias for included studies

Author	Journal	Year	Sample of patients					Exposure		Outcome	
			Eligibility criteria defined	*Sample selection explained (setting, locations, periods of recruitments)*	*Demographic socio-economic characteristics fully described (maternal age, maternal education, family's SES, rural/urban residence, etc)*	*Representativeness of study population*	*Completeness (of the infants eligible for the study, how much proportion (%) were included)*	*Methods to collect exposure variables well defined*	*Exposure assessor blinded to outcome status*	*Methods to collect outcome variables well defined*	*Outcome assessor blinded to exposure status*
BD Schoub	British Medical Journal	1991	good	good	N/A	good	N/R	good	N/A	good	N/A
D. Coetzee	Bulletin of the WHO	1993	good	good	good	good	93.5%	good	N/A	good	N/A
A.Roth	The Pedriatric Infectious Disease	2004	good	good	good	good	N/R	poor	N/A	good	N/A

	Journal										
P.K BORUS	East African Medical Journal	2004	poor	good	poor	poor	N/R	poor	NA	good	NA
A.Jahn	Tropical Medicine and Internatio nal Health	2008	good	good	good	good	100%	good	N/A	good	N/A
A.Sadoh	Public Health	2008	good	good	N/R	poor(only children coming to certain clinic)	N/R	good	N/A	good	N/A
A. Sadoh	Journal of Health Populatio n and Nutrition	2009	good	good	N/R	poor(only children coming to clinic)	N/R	good	N/A	good	N/A

B. Olusanya	Health Research Policy and Systems	2010	good	good	good	good	N/R	good	N/A	good	N/R
JC Moïsi	Vaccine	2010	good	good	poor	good	86.6%	good	NA	good	NA
L.Fadnes	Vaccine	2011	good	good	good	good	86.3%	good	N/A	good	N/A
B. Adebayo	Vaccines and Vaccination	2012	good	poor(no period recorded)	good	good	N/R	good	N/A	good	N/A
J. Babirye	PLoS ONE	2012	good	good	good	good	91.2%	good	N/A	good	N/A
A. Sadoh	Tanzania Journal of Health Research	2013	good	good	good	poor	N/R	good	N/A	good	N/A
O. Waroux	Internaitonal health	2013	good	good	poor	good	N/R	good	N/A	good	N/A
A. Schoeps	Vaccine	2013	good	good	good	good	77.3%	good	N/A	good	N/A

Kidane	East African Medical Journal	2013	good	good	good	good	N/R	good	NA	good	NA
L. Calhoun	Tropical Medicine and Hygiene	2014	good	good	good	poor(study number small?)	N/R	good	N/A	good	N/A
D. Laryea	BMC public health	2014	good	good	poor	poor(only children attending 1 clinic)	N/R	good	N/A	good	N/A
L. Gram	Tropical Medicine and Internatio nal Health	2014	good	good	good	good	66.3%	good	N/A	good	N/A
S. Thysen	BMC public health	2014	good	good	good	good	100%	good	N/A	good	N/A
A. Sadoh	African Health Sciences	2014	good	poor	poor	poor(small population size)	N/R	good	N/A	good	N/A

Lutawama	The Journal of Infectious Diseases	2014	good	good	poor	poor	100%	good	NA	poor	NA
Wagner Z	Vaccine	2014	good	good	poor	poor	100%	good	NA	good	NA
A. Odutola	BMC Health Services Research	2015	good	good	good	good	N/R	good	N/A	good	N/A
D. Gibson	Vaccine	2015	good	good	poor	good	63.9%	good	N/A	good	N/A
R. Miyahara	Vaccine	2016	good	good	good	good	N/R	good	N/A	good	N/A
C. Hoest	Vaccine	2017	good	poor	good	good	N/R	good	N/A	good	N/A
D. Gibson	Lancet Global Health	2017	good	poor	good	good	79.3%	good	no	good	N/A
M. O'Leary	Archives of diseases in childhood	2017	good	good	good	good	96.8%	good	N/A	good	N/A
A. Schweitzer	Bulletin of WHO	2017	good	N/A	N/A	good	N/R	poor	N/A	poor	N/A

P. Zivich	Maternal Child Health Journal	2017	good	poor	good	good	N/R	good	N/A	good	N/A
